# Multifunctional Metal Composite Hydrogels for Diabetic Wound Therapy

**DOI:** 10.3390/gels11120960

**Published:** 2025-11-28

**Authors:** Shengnan Zhang, Hui Gao, Kevin H. Mayo, Jingang Mo, Le Deng

**Affiliations:** 1 College of Life Science, Changchun Normal University, Changchun 130032, China; 2 Department of Biochemistry, Molecular Biology and Biophysics, University of Minnesota, Minneapolis, MN 55455, USA; 3 Department of Microbiology, College of Life Science, Hunan Normal University, Changsha 410081, China; 4 Hunan Provincial Key Laboratory of Microbial Molecular Biology, College of Life Science, Hunan Normal University, Changsha 410081, China

**Keywords:** metal composite hydrogels, diabetic wounds, antibacterial mechanisms, tissue regeneration, clinical translation

## Abstract

Diabetic wounds represent a significant clinical challenge due to a complex pathological microenvironment marked by hyperglycemia, persistent inflammation, and high susceptibility to infection. Metal composite hydrogels have emerged as a next-generation therapeutic platform that uniquely combines the multifaceted capabilities of metal components, such as potent antibacterial, anti-inflammatory, and antioxidant activities, with the ideal moist healing properties of hydrogels. This review focuses on recent progress in the design strategies for these materials, including physical, chemical, and hybrid doping methods. The review then details their multimodal mechanisms of action, including direct antibacterial pathways, like reactive oxygen species generation and photothermal therapy, along with immunomodulatory interventions that regulate macrophage polarization and resolve chronic inflammation. Furthermore, their role in promoting tissue repair processes, such as angiogenesis and collagen remodeling, is discussed. Finally, the review critically examines prevailing challenges and future directions concerning biosafety, scalable fabrication, and clinical translation, with the goal of providing a comprehensive reference for advancing novel therapies for diabetic wound care.

## 1. Introduction

Diabetes is a global chronic metabolic disease and a major public health challenge in the 21st century [1,2]. According to the International Diabetes Federation (IDF) Diabetes Atlas (11th edition), approximately 589 million adults (20–79 years) were living with diabetes globally in 2024, corresponding to ~11.1%. This number is projected to rise to 853 million by 2050. Epidemiological studies reveal that diabetes reduces life expectancy [3]. The global mortality burden is profound, with one death occurring every 9 s, and the disease’s mortality rate has now surpassed the combined mortality rates of acquired immunodeficiency syndrome (AIDS), tuberculosis, and malaria [4,5,6].

Diabetes is primarily classified into two main forms. Type I diabetes (T1D) that accounts for 5–10% of cases, is an organ-specific autoimmune disorder characterized by selective destruction of pancreatic *β*-cells [7]. In contrast, Type II diabetes (T2D) constitutes over 90% of cases and features a heterogeneous pathogenesis, with its core pathophysiological hallmarks being insulin resistance and progressive functional failure of *β*-cells [8,9]. Although their etiologies and management differ, both types share a common pathological basis for complications arising from persistent hyperglycemia, such as diabetic foot, an elevated risk of infection, and diabetic retinopathy [10,11,12]. Among these, the management of diabetic wounds is particularly challenging. It is estimated that 15–25% of diabetic patients will develop chronic, refractory wounds, facing a several-fold times greater risk of infection than the general population [13]. The triad of immune dysfunction, microangiopathy, and peripheral neuropathy, all induced by chronic hyperglycemia, creates a vicious cycle that impairs wound healing [14,15]. This leads to a persistently deteriorating wound microenvironment, heightened susceptibility to secondary bacterial infections, and the establishment of chronic inflammation. Globally, a diabetic limb amputation occurs every 20 s, with uncontrolled wound infections being the primary driver in 85% of these cases.

Normal wound healing is a well-orchestrated process that progresses *via* sequential, overlapping phases: hemostasis, inflammation, proliferation, and remodeling [16]. Hemostasis begins with platelet aggregation and the release of growth factors. This is followed by an inflammatory phase, during which neutrophils and macrophages clear pathogens and debris. Subsequently, the proliferation phase involves collagen deposition by fibroblasts, angiogenesis, and re-epithelialization. Finally, remodeling entails collagen reorganization and scar maturation, typically completing the process within weeks. In contrast, diabetic wounds are severely impaired by hyperglycemia and frequently stagnate in a prolonged inflammatory state [17,18,19]. This dysregulation is characterized by impaired macrophage polarization, excessive production of pro-inflammatory factors such as tumor necrosis factor-α (TNF-α) and interleukin-6 (IL-6), and accumulation of reactive oxygen species (ROS) [20,21,22]. Consequently, fibroblast and keratinocyte functions are suppressed, leading to reduced collagen deposition and impaired angiogenesis. These pathological changes, often compounded by drug-resistant bacterial infections, collectively result in chronic non-healing and increase the risk of gangrene [23].

Current clinical management of diabetic wounds usually involves a multimodal approach, e.g., blood glucose control, surgical debridement, decompression, application of functional dressings (e.g., hydrogels, silver-based dressings), antibiotic therapy, negative pressure wound therapy, hyperbaric oxygen therapy, and the use of growth factors or stem cell therapy [24,25,26,27,28,29,30]. However, the efficacy of these strategies is increasingly challenged by the rise in drug-resistant bacteria, the persistence of biofilms, and particularly the limited efficacy in treating wounds with co-existing vascular complications. Consequently, there is a pressing need to develop novel integrated therapeutic strategies that synergize potent antibacterial activity with effective pro-regenerative functions.

**Figure 1:**
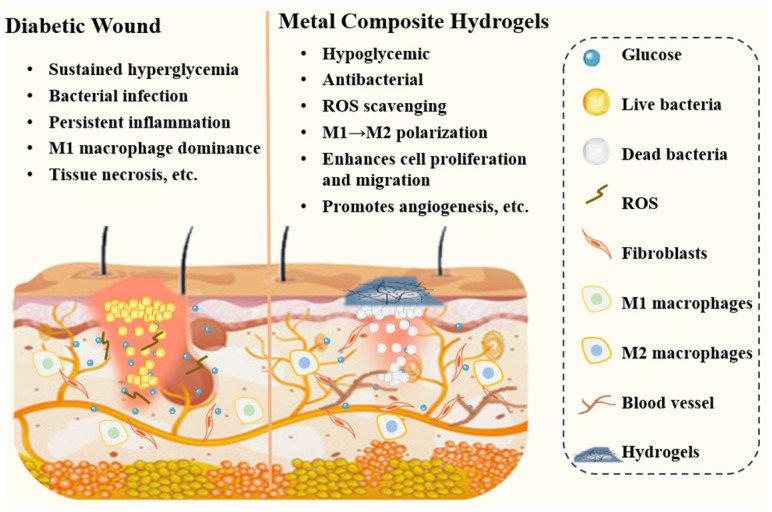
Metal composite hydrogels for the treatment of diabetic wounds.

Hydrogels are gaining increasing attention as diabetic wound dressings. Their inherently porous structure and high water content allow oxygen permeation while maintaining a moist environment, thereby promoting wound healing. However, single-component hydrogel dressings often exhibit certain limitations in antimicrobial efficacy and the promotion of wound healing. Metal composite hydrogels are emerging as multifunctional materials for diabetic wound treatment. They incorporate antimicrobial metals (e.g., silver, copper, zinc) into hydrogel matrices, thereby integrating sustained antibacterial activity with the maintenance of a moist wound environment [31,32]. Research indicates that these materials can effectively eliminate multidrug-resistant bacteria, regulate the wound microenvironment, promote angiogenesis and epithelial regeneration, thus providing a novel therapeutic strategy for chronic diabetic wounds (Figure 1).

This article systematically reviews recent advances in metal composite hydrogels for diabetic wound therapy that includes their composition, fabrication methods, therapeutic efficacy, and underlying mechanisms in both in vitro and in vivo studies. The review also discusses future research directions, with the overall goal of providing a valuable resource for guiding clinical practice and inspiring the development of novel therapeutic materials.

## 2. Preparation of Metal Composite Hydrogels

The hydrogel matrix is typically synthesized from either natural polymers, such as chitosan, collagen, gelatin, sodium alginate, agarose, and hyaluronic acid, or synthetic polymers, including polyethylene glycol (PEG), polyvinyl alcohol (PVA), polyurethane (PU), polylactic acid (PLA), poly(lactic-co-glycolic acid) (PLGA), and polycaprolactone (PCL) [33,34,35,36,37,38]. Hybrid hydrogels that combine natural and synthetic polymers can be engineered to achieve superior performance. The incorporated metal components are diverse, encompassing metallic elements (both single and multi-metal), metal compounds, and metal–organic composites. The fabrication of hydrogels leverages interdisciplinary technologies, including three/four-dimensional (3D/4D) printing, microfluidics, and electrospinning, to achieve functionalization and smart capabilities [39]. Based on the incorporation strategy of the metal-based materials, these composite hydrogels are categorized into three types: physical doping, chemical doping, and hybrid doping (Table 1).

### 2.1. Physically Doped Composite Hydrogels

Metallic materials are physically doped into the hydrogel network *via* non-covalent interactions, such as π-π stacking, hydrogen bonding, electrostatic interactions, and simple blending. This approach features mild processing conditions, operational simplicity, excellent retention of the intrinsic properties of both the metal nanomaterials and the hydrogel, high functional designability, and broad universality [40,49,50]. However, it also presents several limitations. For instance, physical doping can easily cause nanoparticle agglomeration and heterogeneous dispersion. Moreover, the weak binding forces between the metal and the polymer network may lead to metal leakage during long-term use, potentially resulting in functional decay and biosafety concerns. Additionally, incorporated nanoparticles may disrupt the hydrogel network, adversely affecting its mechanical properties and swelling behavior. Therefore, this strategy is best suited for short-term applications where high stability and leakage prevention are not critical.

Despite these limitations, optimizing material composition and interactions in physically doped hydrogels have achieved significant results in diabetic wound treatment. Physical doping of nanomaterials may affect gelation time and mechanical properties of the hydrogels. Chen et al. reported doping Ebselen-loaded dendritic zinc oxide nanoparticles (Ebs@dZnO NPs) into a hyaluronic acid hydrogel, a formulation that delayed drug release and acted synergistically with the ROS that was generated by ZnO and Zn^2+^ even though UV absorption of ZnO prolonged gelation time [51]. However, Gao et al. constructed a polydopamine-coated ZIF-8 (ZIF-8@PDA) nanoparticle composite chitosan hydrogel (COG-Z@P200) that showed enhanced crosslinking and elastic modulus with incorporation of ZIF-8@PDA nanoparticles. This formulation eradicated Methicillin-resistant *Staphylococcus aureus* (MRSA) *via* Zn^2+^-mediated membrane disruption and photothermal effects [52]. In another study, Hu et al. loaded lignin-silver nanoparticles (lignin-Ag) into a chitosan-sodium alginate hydrogel that significantly improving mechanical strength, antibacterial efficacy, and conductivity. It also promoted adhesion, as well as antioxidant and anti-inflammatory properties by formation of quercetin-melanin nanoparticles [53].

Appropriate water absorption plays a crucial role in the therapeutic efficacy of hydrogels, and physical doping of hydrogels can regulate water absorption. For example, Aldakheel et al. used garlic extract for green synthesis of silver nanoparticles (Ag NPs, 50–100 nm) and doped them into a starch-chitosan-alginate free radical polymerization hydrogel, achieving sustained release of Ag NPs and concentration-dependent antibacterial activity, with a 24% improvement in swelling performance [41]. Zhang et al. introduced strontium-doped ZIF-8 (Sr-ZIF-8) into a gelatin methacryloyl (GelMA) hydrogel. These nanoparticles enhanced hydrophobic interactions, which led to water loss and reduced swelling, ultimately resulting in a more stable polymer network structure. The released Zn and Sr ions produced antibacterial and immunomodulatory effects [54].

Physically doped hydrogels can enhance therapeutic efficacy and biological safety. For example, Qi et al. embedded hemoglobin-coated zeolitic imidazolate (Hb@ZIF-8) nanoparticles into a UV-initiated hydrogel that was co-polymerized with silk fibroin methacrylate (SFMA), 3-(acrylamido) phenylboronic acid (AAPBA), and quaternized chitosan (QCS). This approach utilized the disintegration of ZIF-8 in the bacterial microenvironment to release hemoglobin that then induced the prodrug to generate ROS, thus achieving in situ sterilization at the infection site [42]. In another approach, Wang et al. combined copper-doped graphitic carbon nitride (g-C_3_N_4_) with black phosphorus (BP) nanosheets *via* electrostatic bonding and π-π stacking, and dispersed the resultant composite in a polyvinyl alcohol-chitosan (PVA-CS) cross-linked matrix. The copper doping served to accelerate the hot electron flow, thereby enhancing the near-infrared photothermal effect [55]. Similarly, Tang et al. encapsulated copper oxide nanoparticles (CuO NPs) within polymer vesicles to produce copper-polymer nanoreactors (CuO@PS), which were then loaded into a Pluronic F127 (PF127) thermosensitive hydrogel. This configuration preserved the high catalytic activity of the CuO NPs while simultaneously mitigating their direct toxicity [43].

### 2.2. Chemically Doped Composite Hydrogels

Metal ions or nanoparticles can be incorporated into hydrogel networks *via* chemical cross-linking, primarily *via* coordination interactions, covalent integration, and in situ reduction/chelation. This incorporation can enhance the hydrogel’s stability, mechanical properties, and functionality. Nevertheless, these strategies differ significantly in their preparation and impact on the material’s intrinsic properties.

#### 2.2.1. Doping Based on Coordination Interactions

This strategy relies on the dynamic and reversible coordination between metal ions and specific ligands (e.g., carboxyl, catechol, or histidine) present on the polymer chains. These metal-ligand complexes act as dynamic cross-links within the network. This not only enhances mechanical strength and resilience of the hydrogel, but it also facilitates sustained release of metal ions. For instance, Zn^2+^ coordination with glycyrrhizic acid (GA) induces self-assembly that results in a shorter gelation time and improved mechanical strength [44]. In a more complex system, a pre-formed tannic acid-Zn coordinated complex has been cross-linked with phenylboronic acid-modified gelatin *via* boronic ester bonds that achieves controlled release of Zn^2+^ and antibacterial pro-healing [56]. It is important to note that the coordination strength must be precisely controlled. For example, excessive Cu^2+^ (e.g., when chelated by the glycyl-histidyl-lysine peptide) can disrupt protein β-sheet structure, thereby inhibiting hydrogel self-assembly [57].

#### 2.2.2. Doping Based on Covalent Integration

In this approach, covalent bonds form in the metal centers (ions, complexes, or nanoparticles) of the hydrogel network, thus ensuring robust structural integrity and uniform distribution. This can be further categorized by the nature of the covalent bond.

**Figure 2:**
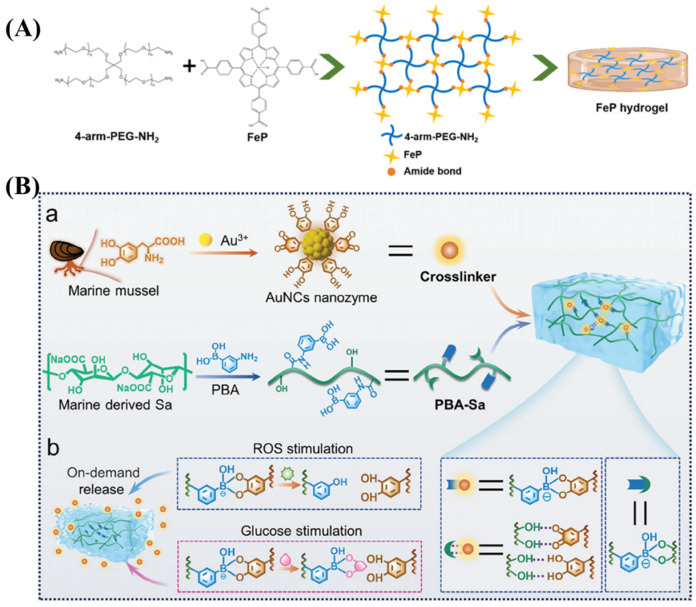
(**A**) Iron-based nanocomposite hydrogels constructed *via* amide bonds [58]. (**B**) Hydrogels prepared *via* dynamic boronic ester bonds enable on-demand release. (**a**) Schematic of preparation and (**b**) release mechanism [59].

Stable covalent bond integration involves permanently anchoring metal-ligand complexes into the network *via* non-reversible bonds. In this case, the complexes primarily serve as functional units rather than the primary structural framework. A prime example is the covalent conjugation of biomimetic catalase (iron porphyrin, FeP) into a PEG network *via* amide bond formation that increases the FeP ratio to increase cross-linking density, reduce swelling ratio, and enhance the modulus, while having a catalytic function (scavenging ROS and producing oxygen) (Figure 2A) [58].

Dynamic covalent bond integration leverages reversible covalent bonds to construct responsive and adaptive networks. For example, gold nanoparticles (Au NPs) surface-riched in catechol groups, form reversible boronic ester bonds with phenylboronic acid-modified sodium alginate (PBA-Sa) that results in a hydrogel with enhanced mechanical properties (stability, elasticity, adhesion), as well as antioxidant and anti-inflammatory activities (Figure 2B) [59]. Similarly, Cu^2+^ form CuPDA nanoparticle complexes containing dopamine have been loaded into hydrogels *via* borate bonds, a formulation that imparts higher cross-linking density, robust adhesion, and photothermal capabilities to the hydrogel [45]. Likewise, the protocatechualdehyde-iron (III) complex (PCA@Fe) can be covalently cross-linked to collagen *via* Schiff base bond formation to enhance the mechanical properties and thermal stability of the hydrogel, as well as integrating the photothermal and antibacterial functions of Fe^3+^ [60].

Thus, whereas stable covalent bonds create a static, high-strength network for sustained functionality, the dynamic covalent bonds introduce stimuli-responsive and self-healing characteristics that collectively advance the design of sophisticated functional hydrogels.

#### 2.2.3. Doping *via* In Situ Reduction and Chelation

This strategy involves the in situ reduction in metal ions to form nanoparticles or nanoclusters, that are simultaneously captured and stabilized within the hydrogel network. This approach combines the high stability of nanomaterials with unique functional properties. For example, hyaluronic acid modified with thiourea groups can reduce Cu^2+^ to mixed-valence copper nanoclusters and chelate/cross-link with them. As the Cu^2+^ concentration increases, cross-linking density increases that leads to reduced swelling. Concurrently, key material properties, such as mechanical strength (reflected in compressive stress) and Young’s modulus, as well as functionalities including photothermal effects and conductivity, are significantly enhanced [46]. In addition, chitosan has been utilized to reduce Ag^+^ in situ that generates Ag NPs. The introduction of PEG further stabilizes the Ag NPs, thus yielding a composite hydrogel with excellent antibacterial and antioxidant properties. However, this process leads to a reduction in water absorption and swelling [61].

Overall, the choice of the chemical doping strategy governs the form of the metal, its distribution uniformity, stability, and the pathway for functional realization. Although these methods may introduce challenges, such as complex preparation and alterations to the hydrogel’s intrinsic properties, the strategic pre-design of molecular groups and composite networks enables a fine balance between stability, mechanical performance, and biological function. This rational design provides strong support for developing advanced functional hydrogels, particularly for applications like smart wound dressings.

### 2.3. Hybrid-Doped Composite Hydrogels

Building on individual doping strategies already discussed, real-world applications often require integration of multiple metals or creation of complex systems. This hybrid approach leverages the strength of both chemical cross-linking and physical interactions. Chemical bonds act as permanent anchors within the network to enhance interfacial binding and prevent metal leakage, while physical interactions preserve functional properties of the metal nano-units by minimizing disruptive chemical modifications. This synergy can lead to formation of inter-penetrating or multiple networks, which improves the hydrogel’s mechanical properties and enables sophisticated functional integration.

**Figure 3:**
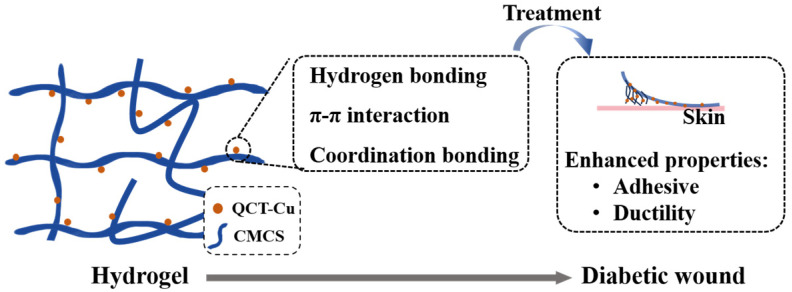
Metal-based nanomaterials incorporated into hydrogels *via* multiple crosslinking mechanisms enable the regulation of their mechanical properties.

In practical systems, the efficacy of this strategy has been demonstrated by both enhanced interfacial interactions and tailored network evolution. For example, nanoparticles formed by coordinated self-assembly of quercetin (QCT) and Cu^2+^ have been embedded into a modified carboxymethyl chitosan (CMCS) hydrogel *via* hydrogen bonding, π-π interaction, and coordination bonding. Although nanoparticle incorporation causes network densification that is manifested in decreased porosity, storage modulus, and swelling rate, it significantly enhances adhesive properties and ductility (Figure 3) [47]. In a porcupine spine-inspired multilayer microneedle system, the copper-doped polyacrylamide hydrogel backing (formed *via* coordination and radical polymerization) enhances the patch’s stability and antibacterial properties. Furthermore, the CaO_2_ nanoparticles and drugs encapsulated within the microneedles, work synergistically to treat diabetic wounds [48].

In conclusion, hybrid doping, by combination of physical and chemical interactions, has shown significant advantages in designing multifunctional hydrogels that are particularly suitable for scenarios requiring high synergy between mechanical properties and biological function. However, its successful implementation depends highly on the fine control of multiple components at the molecular and nanoscale levels. Future efforts, therefore, are required to focus on improving controlled preparations and structural stability of these relatively complex systems.

## 3. Use of Metal Composite Hydrogels in Diabetic Wound Therapy

Diabetic wound healing disorders are mainly manifested in persistent chronic inflammation in a hyperglycemic microenvironment, susceptibility to infection, impaired angiogenesis, and delayed tissue regeneration. Metal composite hydrogels offer a multifunctional synergistic strategy by combining the functional properties of metal components (e.g., antibacterial, anti-inflammatory, angiogenesis-promoting, and immunomodulatory effects) within the three-dimensional hydrophilic structure of the hydrogel matrix. Based on the type of metal used (Table 2), these hydrogels can be categorized as shown in the following sections.

### 3.1. Silver-Based Composite Hydrogels

**Figure 4:**
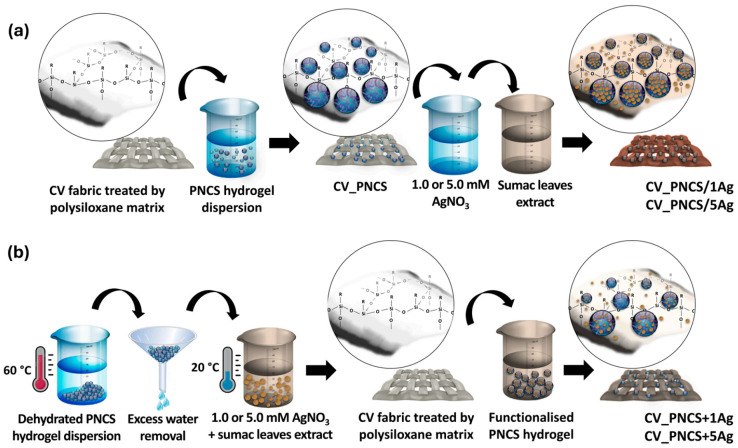
Ag NPs loaded into the PNCS (poly-(N-isopropylakrylamide)/chitosan) hydrogel enabled controlled release. (**a**) In situ synthesis and (**b**) pre-embedding of Ag NPs [73].

Silver-based materials (primarily Ag NPs) are highly useful in diabetic wound therapy. These materials offer broad-spectrum antibacterial activity, a low tendency to induce bacterial resistance, and the capacity to indirectly improve the microenvironment by controlling infections [71,72]. Their antibacterial mechanism of action involves a multi-level process in which Ag^+^ disrupts bacterial cell membranes, interfers with DNA replication, and inactivates functional proteins. In addition, Ag NPs can further enhance efficacy *via* promoting physical contact and inducing localized oxidative stress. Nevertheless, the non-specific biological activity of Ag^+^/Ag NPs at high concentration can lead to cytotoxic effects on normal cells. When incorporated into the polymer network *via* covalent bonding or physical encapsulation, these materials have shown controlled release that provides sustained antibacterial protection and mitigates cytotoxicity (Figure 4) [73].

To enhance antibacterial activity in complex infectious microenvironments, research on silver-based composite hydrogels has focused on sophisticated design strategies that mitigate agglomeration, ensure uniform distribution, and enable sustained release of the nanoparticles. This has been exemplified in a system where Ag NPs and insulin are co-loaded into mesoporous polydopamine nanoparticles (mPD NPs) to form a fibrous hydrogel within a photochemically crosslinked polycaprolactone/methacrylated hyaluronate aldehyde network. These mPD NPs act as carriers to stabilize the Ag NPs, thereby enhancing their overall bacteriostatic performance [62]. Beyond that, a hydrogel based on the polysaccharide ulvan has been constructed *via* a Schiff base reaction and in situ reduction in Ag^+^ that achieves a uniform distribution of Ag NPs and broad-spectrum antibacterial activity [74]. Another method employs green synthesis with *Clerodendrum glandulosum* extracts to produce Ag NPs, that are loaded into a chitosan-PEG hydrogel to achieve sustained release for up to 7 days, along with significant in vitro antibacterial activity [63]. These studies reflect the trend towards synergistic, uniform, and long-lasting antibacterial designs in silver-based hydrogels.

Silver-based materials typically require effects from other functional agents to modulate the wound microenvironment and promote tissue regeneration to accelerate diabetic wound healing. For example, in a T2DM mouse model, aminated Guar Gum/Ag NPs/alginate composite hydrogels (HG-Ag-EGCG) have leveraged the antioxidant epigallocatechin gallate (EGCG) component to efficiently scavenge ROS that significantly reduces oxidative damage and accelerates wound closure [64]. Similarly, the thermo-responsive hydrogel (PAcN/Ag@Clay-TA) promotes antibacterial, antioxidant and anti-inflammatory effects that induce macrophage polarization to the M2 type, and, combined with temperature-triggered contraction facilitates release of nanoparticles to enhance the antibacterial effect and to actively promote diabetic wound closure [75]. Correspondingly, phosphate-responsive hydrogels (PAA-CaP@AgNps@exo) significantly enhance proliferation, migration, and angiogenic capacity of keratinocytes and endothelial cells in a high-glucose environment *via* the action of exosomes and antibacterial Ag NPs. Exosomes play a crucial role in wound healing by regulating inflammation, promoting angiogenesis, and stimulating epithelial cell regeneration. However, their therapeutic potential has been limited by a short half-life and rapid clearance. Hydrogels address these issues by protecting exosomes from premature degradation and enabling their sustained release, thereby achieving their therapeutic effect [76]. In a rat diabetic burn model, a collagen-based composite scaffold (Coll/OJFSP/Ag@Cat) has been shown to regulate signaling pathways (downregulating TGF-*β*1, upregulating TGF-*β*3) that promote scarless healing and skin regeneration [77]. These advances indicate that the function of silver-based hydrogels has evolved from passive antibacterial action to actively regulating cell behavior and regenerative signaling pathways. This, in turn, has provided the possibility to achieve dual structure-function-based repair.

### 3.2. Zinc-Based Composite Hydrogels

Zinc-based composite hydrogels hold great promise for diabetic wound healing due to the multifaceted bioactivities of Zn^2+^ that exerts broad-spectrum antibacterial effects by disrupting bacterial membranes and inhibiting metabolic enzymes. Furthermore, zinc suppresses pro-inflammatory factors, like TNF-*α* and IL-6, thus alleviating chronic inflammation. Additionally, Zn^2+^ inhibits expression of the pancreatic zinc transporter (ZnT8), that helps regulate immune responses and restores islet function [78]. These actions, combined with its ability to promote fibroblast proliferation, collagen deposition, and angiogenesis, allow Zn^2+^ to foster a regenerative microenvironment that accelerates wound closure and remodeling. Zn-based materials are incorporated into hydrogels *via* coordination or physical blending. Among these, dynamic coordination is particularly advantageous, as it enables stable Zn^2+^ loading, while endowing the hydrogel with smart properties [65].

In recent years, ZIF-8-based nanocomposite hydrogels have been widely used for loading functional molecules to enhance therapeutic effects. For example, dihydroquercetin (DHQ) has been loaded into ZIF-8 to form DZ nanoparticles that were embedded into a PVA/graphene oxide/sodium alginate composite hydrogel. This has resulted in DZP hydrogels that exhibit excellent antioxidant, antibacterial, anti-inflammatory, and pro-cell migration properties, and it significantly accelerates diabetic wound healing in mice by inhibiting activation of the IkB*α*/NF-*κ*B pathway [79]. Similarly, a sprayable methacrylic anhydride-modified gelatin (GelMA) hydrogel has been loaded with gcz nanoparticles, a neutrophil-mimicking dual-enzyme system with glucose oxidase (GOx) and chloroperoxidase co-encapsulated in ZIF-8. This hydrogel possesses good sprayability, blue light curability, and biocompatibility that promotes fibroblast proliferation. The gcz nanoparticles consume glucose in wounds to generate HClO, thereby lowering local blood glucose and promoting antibacterial effects. Animal experiments have shown that this hydrogel significantly accelerates wound healing in type I diabetic rats and achieves scarless repair by regulating TGF-*β* expression [80].

Furthermore, zinc-based composite hydrogel response systems have been widely employed in treating diabetic wounds. The dual-cross-linked hydrogel SA-TP@SF@Zn-EA-PL, that is based on a zinc-ellagic acid metal–organic framework (Zn-EA) and *ε*-polylysine (*ε*-PL), integrates glucose-responsive dynamic covalent bonds with static covalent bonds. This design enables an intelligent response to the wound microenvironment and antibacterial-promoting repair, while the photothermal effect of Zn-EA provides mild thermal stimulation that promotes chronic wound healing [66]. In terms of the intelligent response and synergistic therapy, the photo cross-linkable hydrogel adhesive ACGG-ZL (formulated from arginine-methacrylated chitosan (Arg-CSMA) and gallic acid-modified gelatin methacryloyl (GA-GelMA) loaded with a Zn^2+^-Laponite nanoclay (Zn@LAP, ZL)) promotes release of Zn^2+^ and NO in inflammatory microenvironments. This multifunctional system exerts antibacterial, antioxidant, anti-inflammatory properties, promotes neurovascular regeneration, and accelerates wound healing by electrostimulation [81]. Furthermore, a pH-responsive hydrogel GO@ZM that is based on a zinc-metformin coordination complex (Zn@Met), has been developed by integrating photopolymerization and hydrogen bond/Schiff base bond cross-linking mechanisms. By leveraging the immunomodulatory properties of oxidized konjac glucomannan and the antibacterial, anti-inflammatory, and pro-proliferative effects of Zn@Met, this hydrogel has significantly accelerated the healing of MRSA-infected diabetic wounds [82].

**Figure 5:**
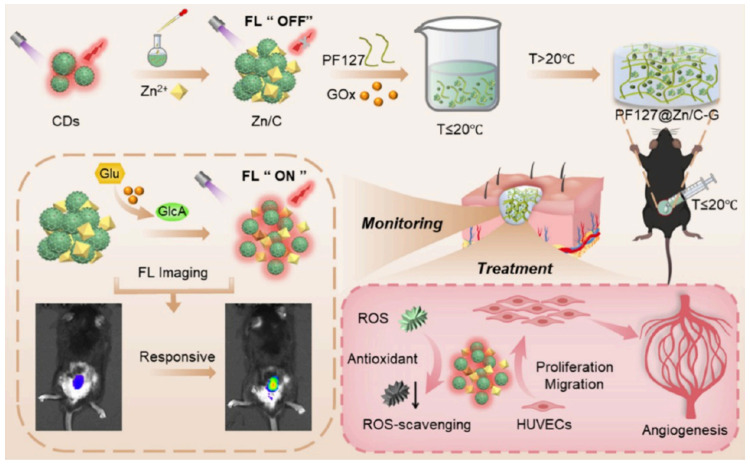
Zinc-based composite hydrogel achieves integration of detection and treatment [83].

Additionally, the dual-layer hydrogel design allows for specialized functions in distinct zones. An antibacterial protective membrane, composed of gelatin methacrylate-alginate-zinc oxide nanoparticles (GelMA-ALG-ZnO), has been combined with a regenerative hydrogel rich in collagen type I and platelet-rich plasma (COL1-PRP). This composition forms a bilayer dressing that forms a physical barrier and has a bioactive function with antibacterial effects from the upper ZnO layer and promotion of cell migration and angiogenesis from the lower layer. In a rat model, this formulation has addressed the complex pathological environment of diabetic foot ulcers and has effectively promoted full-thickness skin defect repair [67]. In another study, an ingeniously designed hydrogel, PF127@Zn/C-G, has been loaded with a Zn/C assembly (formed by the coordination of carbon dots (CDs) with Zn^2+^) and GOx that triggers fluorescence recovery of the CDs *via* GOx-catalyzed acid production from glucose for real-time monitoring of blood glucose levels. In a diabetic mouse model, this integrated platform has promoted the antioxidant and ROS scavenging ability of CDs with the proangiogenic properties of Zn^2+^ to achieve combined blood glucose monitoring and wound therapy (Figure 5) [83]. In a separate bilayer hydrogel system, the inner layer was formed by Schiff base cross-linking of oxidized hyaluronic acid and carboxymethyl chitosan that was loaded with GOx@ZIF-8 to catalyze glucose to produce H_2_O_2_ and an acidic microenvironment which triggers ZIF-8 decomposition and release of antibacterial Zn^2+^. The outer chitosan/PVA hydrogel encapsulates 3,3′,5,5′-tetramethylbenzidine (TMB) and horseradish peroxidase (HRP) for visual quantification of glucose by H_2_O_2_-induced color development. In a diabetic rat infection model, this system combines adhesion, biocompatibility, and antibacterial properties to achieve wound healing within 13 days and integrates glucose monitoring with wound repair [84].

In short, zinc-based composite hydrogels, produced by using diverse materials and functional integration, not only has played a key role in antibacterial, anti-inflammatory, and pro-repair functions, but also has made breakthroughs in intelligent responses, dynamic monitoring, and immune regulation. This, in turn, has provided increasingly comprehensive and innovative solutions for diabetic wound therapy. Nevertheless, several challenges remain: (1) the narrow therapeutic window of zinc ions where excessive release can induce cytotoxicity; (2) the potential compromise of the hydrogel’s mechanical properties with the addition of zinc-based materials; (3) unknown long-term in vivo safety of these complex composite systems; and (4) cumbersome and costly manufacturing processes that hinder clinical scalability.

### 3.3. Copper-Based Composite Hydrogels

Copper-based composite hydrogels offer a highly promising strategy for treating diabetic wounds. The Cu^2+^ released from these hydrogels has multiple bioactive functions, including inherent antibacterial, pro-angiogenic, and immunomodulatory properties. Similarly to silver-based materials, Cu^2+^ exhibits dose-dependent cytotoxicity, primarily by inducing intense oxidative stress *via* Fenton-like reactions and disrupting intracellular metal homeostasis. Therefore, the primary challenge in applying these materials lies in controlling the Cu^2+^ concentration, as excessive release of this ion may be cytotoxic.

Recent advances in material design have effectively mitigated the cytotoxicity issue of Cu^2+^ by stabilization and controlled-release strategies. For instance, the self-assembling hydrogel HLQMes/Cu coordinates L-histidine derivatives and Cu^2+^, which not only significantly reduces ion toxicity, but also promotes self-healing, biocompatibility, and degradability. The optimized HLQMes/Cu1.3 formulation has demonstrated enhanced mechanical strength with a storage modulus of up to 693.7 Pa due to increased cross-linking. In a diabetic mouse model, these hydrogels have achieved a 100% bactericidal effect against *S. aureus* and *E. coli* within 9 h *via* sustained Cu^2+^ release, while simultaneously downregulating pro-inflammatory IL-6, promoting angiogenesis, and facilitating near-complete wound closure by day 11 [85]. Similarly, the H-HKUST-1 system encapsulates a copper metal–organic framework within a thermosensitive antioxidant hydrogel (PPCN), that protects nanoparticles from degradation and ensures sustained Cu^2+^ release. This, in turn, reduces cytotoxicity while promoting keratinocyte and fibroblast migration. In a diabetic mouse model, this system has accelerated wound closure within 21 days by upregulating vascular endothelial growth factor (VEGF) and creating an antioxidant microenvironment [86]. However, its long-term biological safety requires further investigation.

Aside from mitigating copper ion toxicity, copper-based hydrogels have actively regulated the immune microenvironment, which is critical for healing chronic diabetic wounds. Cu-HHA/PVA@MΦ2 hydrogels exemplify this advanced capability. By cross-linking with a low concentration of Cu^2+^, researchers have discovered that this hydrogel serves as a carrier for M2 macrophages and promotes polarization of pro-inflammatory M1 macrophages to the restorative M2 state, thereby alleviating immune suppression. The release of Cu^2+^ works with the hyaluronic acid component to stimulate VEGF secretion, promote angiogenesis, and downregulate TNF-*α*, thus ultimately improving the hypoxic wound microenvironment. In a diabetic mouse model, this immunomodulatory strategy has achieved a wound area reduction of 6.7% in 12 days [87].

Furthermore, integration of Cu^2+^ with other therapeutic modalities has enabled powerful synergistic effects, particularly in managing infected wounds. The PDA-rGO@Cu/CS hydrogel combines the photothermal properties of polydopamine-reduced graphene oxide with the ability of Cu^2+^ to catalyze release of endogenous nitric oxide (NO). In diabetic rats, this combination has yielded a material with potent antibacterial activity under NIR irradiation and continuous NO release for angiogenesis promotion, resulting in 91.57% wound healing after 14 days [88]. In another system, the CMCS/THB/Cu/GB hydrogel utilize Cu^2+^ for antibacterial action and leveraged photothermal effects to stimulate NO release. This multifaceted approach provides antioxidant and anti-inflammatory benefits, significantly downregulates TNF-*α*, promotes collagen deposition, and accelerates wound closure, all supported by self-healing properties that ensure durability in the dynamic wound environment [68].

### 3.4. Iron-Based Composite Hydrogels

Iron-based composite hydrogels represent a novel therapeutic strategy for diabetic wound healing, because they integrate antimicrobial, catalytic, immunomodulatory, and oxygen-regulating effects. These materials catalyze Fenton-like reactions to generate bactericidal ROS from H_2_O_2_, while mimicking catalase activity to decompose H_2_O_2_ into O_2_. In turn, this has alleviated hypoxia and promoted tissue oxygenation. Additionally, these gels promote inflammation resolution and facilitate tissue remodeling by converting macrophages from pro-inflammatory M1 to reparative M2 phenotypes. Iron-based components have typically been incorporated into hydrogels *via* coordination bonds or physical encapsulation, to ensure controlled release while maintaining robust mechanical properties and biocompatibility. This multifunctional design has been particularly well suited for the diabetic wound microenvironment that is characterized by severe oxidative stress and hypoxia. Despite these promising features, the potential toxicity and long-term biosafety of iron necessitate precise dosage control to avoid adverse effects.

Fe^3+^ can be incorporated into injectable PG-Fe hydrogels *via* coordination interactions to produce rapid gelation (11.5 s), superior mechanical properties, strong tissue adhesion (50.5 kPa), self-healing capability (60 s), and degradability. This hydrogel has demonstrated significant antioxidant and anti-inflammatory activities, promoted cell adhesion, migration, and proliferation, and regulated macrophage polarization to the M2 phenotype. Furthermore, this hydrogel can be dissolved and removed on demand using a deferoxamine solution, demonstrating its potential for chronic diabetic wound treatment [89]. Additionally, Fe^3+^ imparts antibacterial properties to the hydrogel by interactions with an antimicrobial agent. Specifically, by coordination between Fe^3+^ and the antimicrobial agent 2,3,4-trihydroxybenzaldehyde (THB), this THB-grafted gelatin forms a multifunctional hydrogel that has avoided the use of strong alkaline reagents or strong oxidizing agents. Hydrogel possesses an adjustable gelation time, excellent rheological properties, and self-healing ability, and exhibits strong adhesion to various materials. Furthermore, the pyrogallol groups endow it with significant antibacterial activity, with a bactericidal rate of ~90% against *S. aureus* and *E. coli*. In a diabetic rat wound infection model, this hydrogel has effectively inhibited infection, reduced inflammatory responses, and promoted wound healing [90].

**Figure 6:**
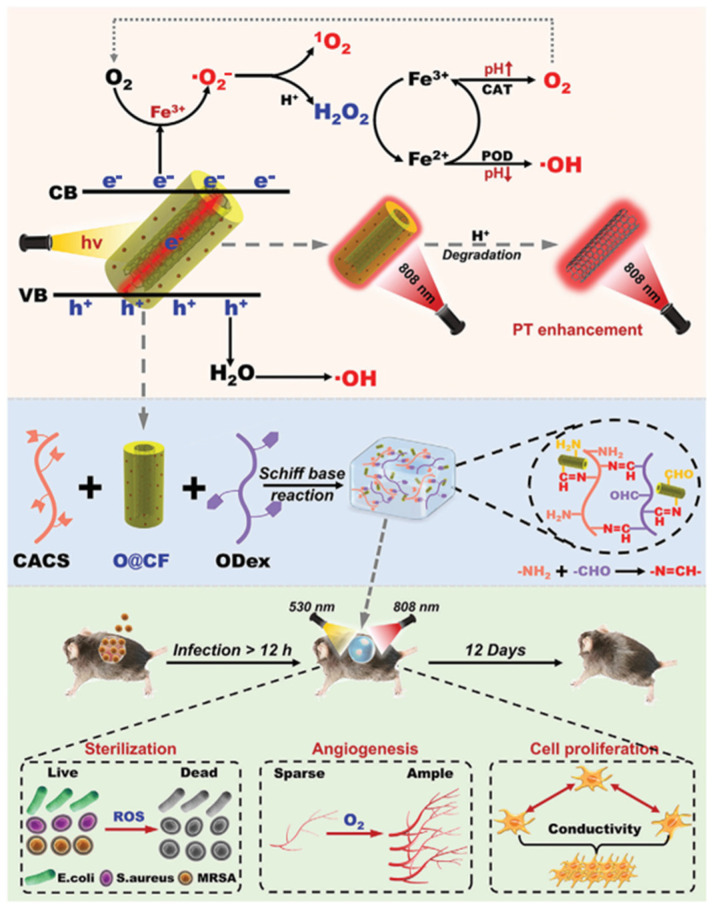
Accelerated healing of diabetic wounds by Fe^3+^ *via* synergistic interactions [91].

In addition, iron-based composite hydrogels have been used in combination to combat bacterial infections and hypoxia in diabetic wounds. The tubular nanocomplex OCNT@COF-Fe (O@CF) has been constructed with a uniform coating of a covalent organic framework (COF) on oxidized carbon nanotubes (OCNTs) and subsequent coordination with Fe^3+^. Within the nanocomplex, OCNT functions as an intermolecular charge-transfer channel, and Fe^3+^ serves as both an electron acceptor and a substrate adsorption site that results in a significant enhancement of the COF’s photodynamic activity for the efficient generation of superoxide anions (·O_2_^−^) and hydroxyl radicals (·OH) under light irradiation. Additionally, O@CF demonstrates pH-dependent peroxidase- and catalase-like activities that convert H_2_O_2_ to·OH under acidic conditions along with its decomposition to O_2_ under neutral conditions, thus alleviating tissue hypoxia. The incorporation of O@CF into the hydrogel not only improves mechanical properties, self-healing capacity, and adhesion, but it also imparts synergistic functionalities, including antibacterial action, biofilm clearance, hypoxia relief, and intercellular electrical signal conduction. These combined attributes result in excellent pro-healing effects in a diabetic wound mouse model infected with methicillin-resistant *Staphylococcus aureus* (MRSA) (Figure 6) [91]. The photothermal-responsive CO_2_@PDA hydrogel is formed by coordinated self-assembly of carboxymethyl *Bletilla striata* polysaccharide (CBSP) and bicarbonate-loaded polydopamine nanoparticles (CO_2_@PDA NPs) with Fe^3+^ into a three-dimensional network. Upon 808 nm near-infrared light irradiation, the CO_2_@PDA NPs generate heat to induce decomposition of HCO_3_^−^ and release of CO_2_. This process alleviates tissue hypoxia *via* the Bohr effect and promotes angiogenesis. Concurrently, CBSP has antioxidant and anti-inflammatory activities, with the action of Fe^3+^ and the photothermal effect delivering potent antibacterial effects. Consequently, this hydrogel has demonstrated efficacy in accelerating the closure of infected wounds in a diabetic rat model [69].

**Figure 7:**
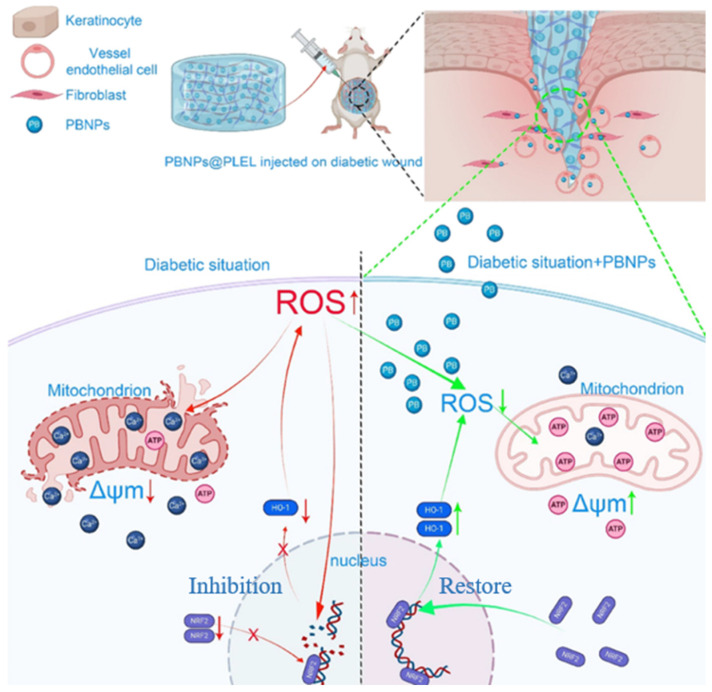
Multi-enzyme-mimetic Fe-based nanocomposite hydrogel accelerates diabetic wound healing [93].

In addition to the use of simple Fe^3+^ ions, other iron-based nanomaterials have also been widely applied for diabetic wound treatment. For instance, a multifunctional hydrogel (GMO@GF) was developed using *Gastrodia elata* polysaccharide (GEP), gelatin methacrylate (GM), and glycyrrhizic acid–iron (GF) nanoparticles. This hydrogel was constructed using a triple-cross-linked structure formed with dynamic Schiff base bonds between oxidized GEP aldehyde groups and GM amino groups, combined with a photo-crosslinked GM network and hydrogen bonding mediated by GF nanoparticles. This integrated design has significantly enhanced mechanical properties, swelling rate, and sustained release capability. In vitro experiments have demonstrated that GMO@GF hydrogels have excellent antibacterial and antioxidant activities, good biocompatibility, and the ability to promote fibroblast and vascular endothelial cell migration. In a diabetic mouse infection model, this hydrogel has significantly reduced inflammation. Simultaneously, it has enhanced angiogenesis and collagen deposition, thereby accelerated wound healing and achieving nearly complete re-epithelialization and tissue repair within 14 days [92]. In in vitro experiments, a thermosensitive hydrogel system (PB NPs@PLEL) based on Prussian blue nanoparticles (PB NPs) has utilized the multi-enzyme mimetic activity of PB NPs to effectively clear ROS and protected keratinocytes, umbilical vein endothelial cells, and fibroblasts from oxidative stress damage, and enhanced mitochondrial functionality. Embedding these nanoparticles into a thermosensitive hydrogel has enabled sustained release of PB NPs and adaptation to different shapes (Figure 7) [93]. Similarly, the PB@GelMA hydrogel has enhanced organic-inorganic interface stability through a pre-coupling strategy that has retained the multi-enzyme activities of PB NPs when combined with far-infrared radiation (~8 μm) emitted by graphene devices. The local temperature rise not only has provided thermal therapeutic effects but has also significantly enhanced ROS scavenging. This combined strategy has promoted diabetic wound healing [94]. Furthermore, an enzyme-crosslinked gelatin-polylysine hydrogel microneedle patch (Gel/PL@Fe^3+^TA), cross-linked by transglutaminase (TGase) catalyzing the reaction between gelatin glutamine residues and polylysine amino groups, has formed a three-dimensional network with adjustable mechanical properties and porosity and has introduced iron-tannic acid nanoparticles (Fe^3+^TA) to impart photothermal responsiveness. Under physiological conditions, this system enzymatically generates ammonia that is metabolized in vivo to NO to promote angiogenesis. This approach has been combined with the endogenous antibacterial ability of polylysine and the photothermal effect of Fe^3+^TA under 808 nm laser irradiation to achieve synergistic bactericidal action against *E. coli* and *S. aureus*. This microneedle patch has significantly accelerated diabetic wound healing, reduced inflammatory factor (TNF-*α*, IL-6) expression, promote collagen deposition and CD31-positive angiogenesis, thus demonstrating good biocompatibility and multiple therapeutic functions [70].

### 3.5. Other Metal Composite Hydrogels

**Figure 8:**
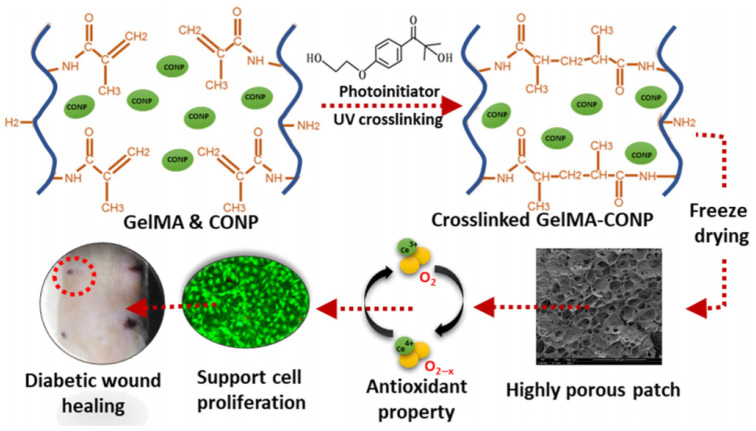
Antioxidant cerium-based composite hydrogels accelerate diabetic wound healing [97].

Aside from the use of metals discussed above, composite hydrogels based on other elements, such as gold, manganese, magnesium, cerium, and their multi-metal combinations, has also demonstrated unique potential in diabetic wound therapy. For example, cerium nanoparticle-based systems have significantly promoted diabetic wound healing through efficient dual antioxidant and anti-inflammatory functions (Figure 8) [95,96,97]. Furthermore, multi-metal synergistic strategies have leveraged complementary interactions between different metal-based materials to achieve enhanced performance in antibacterial activity, anti-inflammation, angiogenesis, and re-epithelialization, thereby overcoming the functional limitations of single-metal systems. These hydrogels are typically fabricated by using multi-component coordination, ion co-doping, or core–shell structural design, to enable stable loading and controlled release of metal components. This approach has provided a critical foundation for the precise and personalized treatment of diabetic wounds.

Several innovative designs have been shown to enhance the clinical use of potential hydrogels. Multiple metal composite hydrogels have demonstrated outstanding performances in improving the hypoxic microenvironment in diabetic wounds. For example, calcium peroxide nanoparticles have been incorporated to release oxygen and alleviate hypoxia [98]. Metal-based hydrogels that integrate MnO_2_ nanoparticles, have effectively catalyzed the conversion of endogenous H_2_O_2_ to O_2_ at wound sites, thereby improving tissue oxygenation. A multifunctional composite hydrogel (SA@ MnO_2_/RHC/MSCs), composed of MnO_2_ nanoparticles, recombinant human collagen III (RHC), and bone marrow mesenchymal stem cells (MSCs), has been shown to exhibit superior efficacy in re-epithelialization, collagen deposition, and angiogenesis by synergistic oxygen generation and stem cell-mediated repair [99]. The catalytic activity of MnO_2_ can be further enhanced with MnO_2_-Au nanoflowers [100]. Moreover, a graphene oxide-based hydrogel loaded with silver-platinum nanoparticles (GO@Ag-Pt) has shown simultaneous ROS scavenging, O_2_ generation, and broad-spectrum antibacterial activity [101]. Notably, a pH-responsive multi-enzyme active nanocomposite hydrogel (Mo,Fe/Cu,I-Ag@GOx) has exhibited multiple enzyme-like activities that have enabled glucose-triggered cascade reactions to generate ROS under acidic conditions and supply O_2_ under alkaline conditions on-demand [102].

Meanwhile, the ROS-responsive hydrogel MSL@Z/G has been constructed by encapsulating ZIF-90 nanoparticles loaded with metformin, strontium ions, and L-Arg into methacrylated gelatin, which has promoted the survival of diabetic multi-territory perforator flaps [103]. Using hollow Cu_2_S nanocubes as a template, an intelligent hydrogel (Cu_2_S@FePPOPₜₚₘ@GOx@Gel) has been constructed *via* in situ growth of FePPOPₜₚₘ and loaded GOx that have enabled release of nanocomposites under acidic pH conditions in infected microenvironments to kill bacteria [104]. A glucose-activated nanozyme hydrogel (Cu-TCPP(Fe)@Au@BSA) has utilized a two-dimensional metal–organic framework loaded with gold nanoparticles, thus forming a dual-active system with GOx and peroxidaselike-like activities to efficiently sterilize and promote healing in high-glucose microenvironments [105]. A pomegranate structure-inspired gold/silver nanodot composite dressing (Au/AgNDs@Gel) has combined fluorescent monitoring, photothermal antibacterial effects, and wound healing [106]. These responsive hydrogels have collectively demonstrated significant potential in advancing personalized treatment strategies for healing diabetic wounds, thus offering a promising direction for clinical use.

In brief, metal composite hydrogels have demonstrated significant potential for multi-mechanistic regulation and personalized application in diabetic wound therapy. Their core advantages include: (1) Multifunctional integration capable of simultaneously conferring antibacterial, anti-inflammatory, antioxidant, pro-angiogenic, and immunomodulatory properties, while improving hypoxic microenvironments; (2) Environmental responsiveness that intelligently respond to changes in wound microenvironmental pH, temperature, glucose, or ROS levels to achieve on-demand drug release to enhance treatment outcome and minimize side effects [107]; (3) Biocompatibility and processability in many systems have been based on the use of natural polymers (e.g., gelatin, sodium alginate, chitosan) that have injectable/self-healing properties to facilitate adaptation to irregular wounds and reduce replacement-related damage [108,109,110].

Nevertheless, the choice of metal critically influences both therapeutic outcome and biosafety. For example, Ag NPs, while potent antibacterial agents, can be cytotoxic when over-accumulated. Essential trace elements Zn and Cu ions (unlike silver) have been shown to boast biocompatibility and promote healing though their antimicrobial activities. Fe-based systems, useful for their enzyme-mimicking antioxidant activities, can promote oxidative stress *via* Fenton reactions. Hence, adopting a balanced approach to metal selection and precise control over its release are crucial for harmonizing efficacy and safety.

In addition to the critical choice of metal, the clinical translation of metal composite hydrogels has been hindered by four major challenges: (1) Long-term safety verification: the prolonged retention in vivo of metal nanoparticles (e.g., Ag, CeO_2_, Fe_3_O_4_) poses toxicity risks due to unclear metabolic pathways and immunogenicity concerns, including local/systemic toxicity from metal ion leaching and nanoparticle accumulation; (2) Preparation complexity and scalability: multi-component nano-composite synthesis involves cumbersome steps (e.g., multi-step coordination, core–shell assembly) with batch-to-batch variability. Scalable production requires optimized quality control protocols to ensure reproducibility and clinical-grade consistency; (3) Efficacy evaluation gaps: current studies predominantly use small animal models, lacking large animal experiments and investigations into chronic disease complexity (e.g., comorbidities, heterogeneous wounds). Clinical-grade validation will require phase I/II trials to confirm safety and efficacy in humans; (4) Cost and stability constraints: precious metal-based systems (e.g., Au, Pt) incur high costs, while nanozyme activities be unstable in complex physiological environments. Stability enhancement *via* surface modification (e.g., polymer coating) and cost reduction through biocompatible alternatives (e.g., Fe-based nanozymes) are critical research directions. Among these, the most critical aspect of clinical translation has been to ensure biological safety.

## 4. Biosafety Evaluation

The safety of wound treatment materials has been a central concern that must be addressed prior to entry into a clinical setting. Metal composite hydrogels, which consist of synthetic/natural polymer matrices and metal-based components, possess complex chemical compositions and structures. This complexity necessitates establishment of a systematic and comprehensive biosafety evaluation system. Such assessments must not only adhere to international standards, but also to fully considering the unique pathological environment of diabetic wounds. Currently, biosafety evaluations are primarily divided into in vitro and in vivo studies that together provide scientific evidence for material safety.

### 4.1. In Vitro Evaluation Methods

In vitro methods have been characterized by high throughput, low cost, and excellent controllability, making them the primary screening tools for biosafety assessment. For metal composite hydrogels, key in vitro evaluations encompass cytotoxicity, hemocompatibility, and effects on specific cellular functions.

Cytotoxicity is a fundamental indicator of biomaterial safety. Cell lines like mouse fibroblasts (NIH 3T3) and human dermal fibroblasts are commonly employed. Cell viability and proliferation are assessed using assays such as the Cell Counting Kit-8 (CCK-8) and the 3-(4,5-Dimethylthiazol-2-yl)-2,5-Diphenyltetrazolium Bromide (MTT) assay, with a relative proliferation rate > 90% that are generally considered non-cytotoxic [111,112]. Hemocompatibility, crucial for hydrogel dressings in direct contact with blood, primarily includes hemolysis and coagulation assays [113,114]. An ideal wound dressing should prevent hemolysis and support rapid hemostasis at the wound site. Aside from cell viability and hemocompatibility, it is essential to evaluate effects of these hydrogels on specific cellular functions that are critical to wound healing, such as cell migration, proliferation, and differentiation [115].

### 4.2. In Vivo Evaluation Methods

In vivo evaluation has provided indispensable and holistic safety information for metal composite hydrogels within a functioning biological system. This has made it a cornerstone to preclinical development. Its scope for diabetic wound dressings encompasses local tissue response, systemic toxicity, and the long-term fate of the materials.

The local oxidative stress and chronic inflammation in diabetic wounds can alter material safety, making assessments in diabetic animal models more predictive. For instance, researchers have employed streptozotocin-induced diabetic mouse models with full-thick skin injuries to evaluate hydrogels. Histopathological analyses in such models have provided insight into long-term tissue effects, offering a more clinically relevant safety profile [116]. Assessment of wound healing is another vital in vivo safety metric that extends beyond merely tracking wound closure rates to include evaluating epidermal thickness, collagen deposition, angiogenesis, and inflammatory responses [117]. Systemic toxicity assessment has focused on potential adverse effects when material components enter the systemic circulation. For metal composite hydrogels, it is essential to investigate whether released metal ions accumulate in various organs (e.g., liver, spleen, kidneys) and cause toxic damage [118]. Finally, long-term biodistribution and metabolic studies have tracked the fate of these materials in vivo, particularly metabolic pathways, levels of accumulation, and clearance of metal nanoparticles, all of which are fundamental for determining long-term safety [119].

## 5. Conclusions, Challenges, and Prospects

Metal composite hydrogels, as a class of emerging multifunctional biomaterials, have shown significant potential for clinical translation for diabetic chronic wound therapy. This article has systematically reviewed various construction strategies, functional mechanisms of action, and preclinical research progress of widely studied metal composite hydrogels. By integrating the multiple biological effects of metal components (antibacterial, anti-inflammatory, antioxidant, immunomodulatory, pro-angiogenic) with the maintenance of a moist environment and controlled release characteristics of hydrogels, these systems have been shown to achieve intervention and synergistic therapy for the complex microenvironment within diabetic wounds (e.g., hyperglycemia, hypoxia, oxidative stress, microbial infection). In particular, the employment of multi-metal strategies and intelligent responsive designs (e.g., ROS, pH, glucose sensitivity) have further enhanced the comprehensive performance of these materials, thereby providing innovative ideas for multi-dimensional therapy of diabetic wounds.

Future research is required to prioritize several key directions for clinical translation of metal composite hydrogels. This includes long-term safety concerns due to prolonged in vivo retention, manufacturing complexities that hinder scalability, a lack of robust efficacy in relevant animal models, and constraints regarding cost and stability. In this regard, the primary focus should be to optimize preparation processes and establish standards. This involves the development of advanced manufacturing technologies, such as green synthesis, microfluidics, and 3D printing, to enable large-scale, standardized production, which is key to achieving superior batch-to-batch consistency and stability. Exploring low-cost alternatives, such as biodegradable metal alloys, is also crucial for reducing clinical application costs.

Regarding intelligent responsiveness and personalized treatments, next-generation metal-hydrogel systems should be engineered to adapt to the heterogeneity of diabetic wounds. This heterogeneity encompasses variations in infection severity, tissue vascularization, and individual metabolic profiles. Ideally, these systems require multi-stimuli responsiveness (e.g., to temperature, enzymes, or electrical signals) and dynamic regulation to achieve precise, personalized treatment. Furthermore, their integration with cutting-edge technologies, like gene editing and cell therapy, could enable the creation of synergistic “material-cell-drug” platforms.

However, significant challenges remain with in vivo studies, particularly concerning safety and metabolic mechanisms of action. Comprehensive studies on in vivo metabolic kinetics, biodistribution, and long-term toxicity of metal ions and nanomaterials are essential. Establishing robust toxicity evaluation approaches that are based on multi-omics approaches (e.g., metabolomics, proteomics) and organoid models (e.g., organs-on-a-chip) will help to clarify safe dosing and exposure risks. Furthermore, large animal models and preclinical research are indispensable. Diabetic wound models in large animals (e.g., pigs, dogs) are needed to validate the efficacy and safety of these materials in large-volume wounds, complex anatomical sites, and extended treatment periods. Multi-center preclinical studies, combined with real-world data, will be critical for evaluating the performance and applicability of hydrogels in clinically relevant environments.

In summary, metal composite hydrogels offer a highly promising strategy for treating chronic diabetic wounds; however, their successful clinical translation relies on interdisciplinary use of materials science, biomedicine, clinical medicine, and other fields. Through systematic and in-depth basic research, process optimization, and clinical validation, these biomaterials can ultimately make the leap from laboratory to clinic, bringing safer, more effective, and accessible treatment options for diabetic wound patients.

## Figures and Tables

**Table 1:** Integration Strategies of Metal-based Materials in Hydrogels.

Doping Method	Cross-Linking Mechanism	Advantages	Disadvantages	References
Physical Doping	Non-covalent interactions (hydrogen bonding, electrostatic interactions, π-π stacking)	Mild preparation conditions; Simple and straightforward procedure; Broad applicability; Excellent retention of intrinsic properties.	Poor dispersion of the metal based materials; Leakage of metal ions or particles; Potential biosafety concerns; Structural disruption of the hydrogel network; Compromised mechanical properties.	[40,41,42,43]
Chemical Doping	Covalent and coordination interactions (coordination bonds, Schiff base bonds, boronic ester bonds, amide bonds)	Enhanced stability; Homogeneous distribution of metal ions/particles; Controlled release of metal ions/particles; Improved mechanical properties.	Complex and demanding preparation process; Altered hydrogel mechanical properties; Potential safety concerns; Limited functional flexibility and reversibility.	[44,45,46]
Hybrid Doping	Combination of physical and chemical methods (e.g., coordination bonds and hydrogen bonding/π-π stacking)	Synergistic structural stability; Enhanced functional diversity; Formation of interpenetrating networks; Superior mechanical properties; Successful functional integration.	Stringent process control and poor reproducibility; Difficulty in achieving interfacial compatibility and uniform distribution; Unpredictable interference between different networks; Increased risk of structural defects; Unpredictable mechanical performance.	[47,48]

**Table 2:** Representative Metal Composite Hydrogels for Diabetic Wound Therapy.

Metal Type	Function Category	Mechanism	Therapeutic Effect	References
Ag	Antibacterial	Disrupts bacterial cell membranes; interferes with DNA replication; denatures proteins	Broad-spectrum antibacterial activity; low risk of resistance	[41,62,63,64]
	Angiogenesis and re-epithelialization	Synergizes with functional molecules (e.g., insulin) to promote cell migration and proliferation	Accelerates wound closure; promotes granulation tissue formation	
	Antioxidant and anti-inflammatory	Scavenges ROS; induces macrophage polarization toward the M2 phenotype	Alleviates oxidative stress and inflammation; improves healing microenvironment	
Zn	Antibacterial and anti-inflammatory	Disrupts bacterial membranes; inhibits metabolic enzymes; downregulates pro-inflammatory factors (TNF-*α*, IL-6)	Alleviates chronic inflammation; effectively clears bacteria	[65,66,67]
	Pro-healing and immunomodulation	Promotes fibroblast proliferation and collagen deposition; regulates macrophage M1/M2 polarization	Accelerates wound closure and tissue remodeling; facilitates scarless repair	
	Smart response/ Theranostics	Responsive release; monitoring of glucose	On-demand therapy; integrates diagnosis and treatment functions	
Cu	Antibacterial	Generates ROS; disrupts bacterial membrane integrity	Highly efficient bactericidal activity; effective against drug-resistant bacteria	[43,68]
	Angiogenesis	Promotes vascular endothelial cell proliferation and migration	Improves ischemic and hypoxic microenvironment; accelerates angiogenesis	
	Immunomodulation	Modulates macrophage polarization; promotes transition to M2 anti-inflammatory phenotype	Alleviates chronic inflammation; promotes inflammation resolution	
Fe	Antibacterial	Fenton-like reaction produces ROS; directly disrupts bacterial cell structure; photothermal effect generates heat	Provides a potent antibacterial effect	[58,69,70]
	Antioxidant	Mimics nanozyme activity to scavenge ROS	Alleviates oxidative stress	
	Anti-inflammatory	Regulates macrophage polarization from M1 to M2 phenotype	Downregulates TNF-*α*, IL-1*β*; upregulates IL-10, TGF-*β*1; alleviates excessive inflammation	
	Oxygenation	Decomposes H_2_O_2_ to release O_2_	Alleviates tissue hypoxia	

## Data Availability

No new data were created or analyzed in this study. Data sharing is not applicable to this article.

## References

[1] Huang J.F., Chang T.J., Yeh M.L., Shen F.C., Tai C.M., Chen J.F., Huang Y.H., Hsu C.Y., Cheng P.N., Lin C.L. (2024). Clinical care guidance in patients with diabetes and metabolic dysfunction-associated steatotic liver disease: A joint consensus. Hepatol. Commun..

[2] Huang R., Jun D.W., Toyoda H., Hsu Y.C., Trinh H., Nozaki A., Ishikawa T., Watanabe T., Uojima H., Huang D.Q. (2025). Impacts of metabolic syndrome diseases on long-term outcomes of chronic hepatitis B patients treated with nucleos(t)ide analogues. Clin. Mol. Hepatol..

[3] Sun H., Saeedi P., Karuranga S., Pinkepank M., Ogurtsova K., Duncan B.B., Stein C., Basit A., Chan J.C.N., Mbanya J.C. (2022). IDF Diabetes Atlas: Global, regional and country-level diabetes prevalence estimates for 2021 and projections for 2045. Diabetes Res. Clin. Pract..

[4] Bronnum-Hansen H., Davidsen M., Andersen I. (2023). Impact of the association between education and obesity on diabetes-free life expectancy. Eur. J. Public Health.

[5] Carlsson L.M.S., Carlsson B., Jacobson P., Andersson-Assarsson J.C., Karlsson C., Kristensson F.M., Ahlin S., Näslund I., Karason K., Svensson P.A. (2025). Association between delay in diabetes development and mortality in people with obesity: Up to 33 years follow-up of the prospective swedish obese subjects study. Diabetes Obes. Metab..

[6] Wang B., Fu Y.Q., Tan X., Wang N.J., Qi L., Lu Y.L. (2024). Assessing the impact of type 2 diabetes on mortality and life expectancy according to the number of risk factor targets achieved: An observational study. BMC Med..

[7] Moreno-Castellanos N., Cuartas-Gómez E., Vargas-Ceballos O. (2023). Functionalized collagen/poly(ethylene glycol) diacrylate interpenetrating network hydrogel enhances beta pancreatic cell sustenance. Gels.

[8] Nimmagadda S.M., Suryanarayana G., Kumar G.B., Anudeep G., Sai G.V. (2024). A comprehensive survey on diabetes Type-2 (T2D) forecast using machine learning. Arch. Comput. Methods Eng..

[9] Harris C., Czaja K. (2023). Can circadian eating pattern adjustments reduce risk or prevent development of T2D?. Nutrients.

[10] Wendland D.M., Altenburger E.A., Swen S.B., Haan J.D. (2025). Diabetic foot ulcer beyond wound closure: Clinical practice guideline. Phys. Ther..

[11] Khan M.S., Jahan N., Khatoon R., Ansari F.M., Ahmad S. (2025). The diabetic foot ulcer: Biofilm, antimicrobial resistance, and amputation. Int. J. Diabetes Dev. Ctries..

[12] Lee J., Mashayamombe M., Walsh T.P., Kuang B.K.P., Pena G.N., Vreugde S., Cooksley C., Carda-Diéguez M., Mira A., Jesudason D. (2023). The bacteriology of diabetic foot ulcers and infections and incidence of *Staphylococcus aureus* small colony variants. J. Med. Microbiol..

[13] Chary P.S., Urati A., Shaikh S., Yadav R., Bhavana V., Rajana N., Mehra N.K. (2025). Nanotechnology-enabled approaches for combating diabetic foot ulcer. J. Drug Deliv. Sci. Technol..

[14] Uddin M.N., Thomas D.W. (2024). High Glucose alters endoplasmic reticulum Ca^2+^ stores in T lymphocytes revealing potential novel therapeutic targets for diabetic-induced immune dysfunction. Physiology.

[15] Liu C.J., Liu K., Zhang D., Liu Y.T., Yu Y.F., Kang H.F., Dong X.Z., Dai H.L., Yu A.X. (2025). Dual-layer microneedles with NO/O_2_ releasing for diabetic wound healing *via* neurogenesis, angiogenesis, and immune modulation. Bioact. Mater..

[16] Wang F., Zhang W.Y., Li H., Chen X.N., Feng S.N., Mei Z.Q. (2022). How effective are nano-based dressings in diabetic wound healing? A comprehensive review of literature. Int. J. Nanomed..

[17] Wang T., Zheng Y., Shi Y.J., Zhao L. (2019). pH-responsive calcium alginate hydrogel laden with protamine nanoparticles and hyaluronan oligosaccharide promotes diabetic wound healing by enhancing angiogenesis and antibacterial activity. Drug Deliv. Transl. Res..

[18] Yan X.J., Zheng W.F., Yu Y.M., Liu R.F., Gong Y.H., Huang M.T., Fan M.D., Wang L. (2025). Dextran-functionalized cerium oxide nanoparticles for treating diabetic wound infections by synergy between antibacterial activity and immune modulation. Mater. Today Bio.

[19] Li X.M., Qu S., Ouyang Q.H., Qin F., Guo J.M., Qin M., Zhang J.J. (2024). A multifunctional composite nanoparticle with antibacterial activities, anti-inflammatory, and angiogenesis for diabetic wound healing. Int. J. Biol. Macromol..

[20] Wu X.Q., He W.J., Mu X.R., Liu Y., Deng J.Y., Liu Y.Q., Nie X.Q. (2022). Macrophage polarization in diabetic wound healing. Burn. Trauma.

[21] Accipe L., Abadie A., Neviere R., Bercion S. (2023). Antioxidant activities of natural compounds from caribbean plants to enhance diabetic wound healing. Antioxidants.

[22] Jebakumar M., Pachaiyappan M., Kamini N.R., Radhakrishnan J., Ayyadurai N. (2025). Engineered spider silk in core-shell multifunctional fibrous mat for accelerated chronic diabetic wound healing *via* macrophage polarization. ACS Biomater. Sci. Eng..

[23] Wang Z., Tan X.Y., Xue Y., Xiao C., Yue K.J., Lin K.B., Wang C., Zhou Q.H., Zhang J.L. (2024). Smart diabetic foot ulcer scoring system. Sci. Rep..

[24] Aybar J.N.A., Mayor S.O., Olea L., Garcia J.J., Nisoria S., Kolling Y., Melian C., Rachid M., Dimani R.T., Werenitzky C. (2022). Topical administration of lactiplantibacillus plantarum accelerates the healing of chronic diabetic foot ulcers through modifications of infection, angiogenesis, macrophage phenotype and neutrophil response. Microorganisms.

[25] Rozen S.M., Wolfe G.I., Vernino S., Raskin P., Hynan L.S., Wyne K., Fulmer R., Pandian G., Sharma S.K., Mohanty A.J. (2024). Effect of lower extremity nerve decompression in patients with painful diabetic peripheral neuropathy. Ann. Surg..

[26] Wang H.W., Wang L.H., Liu Z.M., Luo Y.M., Kang Z.C., Che X. (2024). Astragaloside/PVP/PLA nanofiber functional dressing prepared by coaxial electrostatic spinning technology for promoting diabetic wound healing. Eur. Polym. J..

[27] Xie Q.C., Wang J.Y., Huang G.F., Dai J.Z. (2025). Silver dressings for treating diabetic foot ulcers: A systematic review and meta-analysis. J. Tissue Viability.

[28] Wang Y.L., Liu B., Pi Y.Z., Hu L., Yuan Y.L., Luo J., Tao Y.X., Li P., Lu S., Song W. (2022). Risk factors for diabetic foot ulcers mortality and novel negative pressure combined with platelet-rich plasma therapy in the treatment of diabetic foot ulcers. Front. Pharmacol..

[29] Ristic P., Savic M., Bolevich S., Bolevich S., Orlova A., Mikhaleva A., Kartashova A., Yavlieva K., Turnic T.N., Pindovic B. (2023). Examining the effects of hyperbaric oxygen therapy on the cardiovascular system and oxidative stress in insulin-treated and non-treated diabetic rats. Animals.

[30] Nieuwland A.J., Waibel F.W.A., Flury A., Lisy M., Berli M.C., Lipsky B.A., Uçkay I., Schöni M. (2023). Initial antibiotic therapy for postoperative moderate or severe diabetic foot infections: Broad versus narrow spectrum, empirical versus targeted. Diabetes Obes. Metab..

[31] Abbasi Y.F., Guo X., Chen Y., Li J.H., Xu X.Y., Li Y.X., Cun D.M., Bera H., Yang M.S. (2025). High molecular weight laminarin/AgNPs-impregnated PVA based in situ hydrogels accelerated diabetic wound healing. Carbohydr. Polym..

[32] Ding J.X., Gao B.B., Chen Z.H., Mei X.F. (2021). An NIR-triggered Au nanocage used for photo-thermo therapy of chronic wound in diabetic rats through bacterial membrane destruction and skin cell mitochondrial protection. Front. Pharmacol..

[33] Peng S.T., Wang L.T., Lu Z.F., Yang X.Y., Lu Y.X., Wang Z.X., Wu Q.X., Qin X.F. (2026). An injectable conductive multifunctional hydrogel dressing with synergistic antimicrobial, ROS scavenging, and electroactive effects for the combined treatment of chronic diabetic wounds. Biomater. Adv..

[34] Zhuang Z.M., Wang Y., Chen L., Wu Z.R., Zhang T., Bei H.P., Feng Z.X., Wang Y., Guo K., Hsu Y.Y. (2026). Triple-molded, reinforced arrowhead microneedle patch of dual human-derived matrix for integrated management of diabetic wounds. Biomaterials.

[35] Zhou Y.L., Liang X.Y., Shen Z.Y., Zhang R., Zhang G., Yu B.R., Li Y., Xu F.J. (2026). Glucose-responsive hydrogel with adaptive insulin release to modulate hyperglycemic microenvironment and promote wound healing. Biomaterials.

[36] Su X.C., Geng X.R., Zhang Y.F., Shi Y.J., Zhao L. (2024). Microenvironmental pH modulating oxygen self-boosting microalgal prodrug carboxymethyl chitosan/hyaluronic acid/puerarin hydrogel for accelerating wound healing in diabetic rats. Int. J. Biol. Macromol..

[37] Dai S.Y., Mao L.C., Chen X.W., Zhang J., Li X., Zhang M.S., Jiang N., Yang K.D., Duan S., Gan Z.H. (2026). A heterogeneous hydrogel patch with mechanical activity and bioactivity for chronic diabetic wound healing. Biomaterials.

[38] Metwally W.M., El-Habashy S.E., El-Hosseiny L.S., Essawy M.M., Eltaher H.M., El-Khordagui L.K. (2024). Bioinspired 3D-printed scaffold embedding DDAB-nano ZnO/nanofibrous microspheres for regenerative diabetic wound healing. Biofabrication.

[39] Wang C.L., Shahriar S.M.S., Su Y.J., Hayati F., Andrabi S.M., Xiao Y.Z., Busquets M.E., Sharma N.S., Xie J.W. (2025). Three-dimensional bioprinting of biphasic nanobioink for enhanced diabetic wound healing. ACS Nano.

[40] Yang L.J., Wang C., Zhang C.Y., Qi W., Wang M.F. (2025). A hydrogen-bonded biohybrid organic framework hydrogel for enhanced diabetic wound therapy. Chem. Eng. J..

[41] Aldakheel F.M., Mohsen D., El Sayed M.M., Fagir M.H., El Dein D.K. (2023). Green synthesized silver nanoparticles loaded in polysaccharide hydrogel applied to chronic wound healing in mice models. Gels.

[42] Qi X.L., Lan Y.L., Chen J., Xiang Y.J., Wang Y.Y., Jiang L.T., Dong Y.J., Li J.X., Liao Z.Y., Li Z.P. (2025). An endogenous adenosine triphosphate-activated hydrogel prodrug system for healing multidrug-resistant bacteria infected diabetic foot ulcers. Adv. Healthc. Mater..

[43] Tang Q., Tan Y.Q., Leng S.L., Liu Q., Zhu L.Y., Wang C.F. (2024). Cupric-polymeric nanoreactors integrate into copper metabolism to promote chronic diabetic wounds healing. Mater. Today Bio.

[44] Li J., Wu D.G., Su Z.W., Guo J.Y., Cui L.Y., Su H., Chen Y., Yu B. (2025). Zinc-induced photocrosslinked konjac glucomannan/glycyrrhizic acid hydrogel promotes skin wound healing in diabetic mice through immune regulation. Carbohydr. Polym..

[45] Zhu S.L., Zhao B.J., Li M.C., Wang H., Zhu J.Y., Li Q.T., Gao H.C., Feng Q., Cao X.D. (2023). Microenvironment responsive nanocomposite hydrogel with NIR photothermal therapy, vascularization and anti-inflammation for diabetic infected wound healing. Bioact. Mater..

[46] Qiu X.A., Xiang F., Bu P.Z., Lv Q.Q., Liu X.Z., Zhou B.K., Tan M.J., Jiang X., Cheng X., Serda M. (2025). A hydrogel crosslinked with mixed-valence copper nanoclusters for diabetic wound healing. Acta Biomater..

[47] Zhu Y., Yang S.Y., Zhou J., Zhang Z.X., Hu Y., Xu Q.Y., Zhou Y.N., Xu Y.J. (2025). An injectable triple-responsive chitosan-based hydrogel encapsulating QCT-Cu self-assembled nanoparticles accelerating diabetic wound healing *via* remodeling the local microenvironment. Carbohydr. Polym..

[48] Liu T.Q., Sun Y.F., Jiang G.H., Zhang W.J., Wang R., Nie L., Shavandi A., Yunusov K.E., Aharodnikau U.E., Solomevich S.O. (2023). Porcupine-inspired microneedles coupled with an adhesive back patching as dressing for accelerating diabetic wound healing. Acta Biomater..

[49] Hao M.K., Wei S.M., Su S.Q., Tang Z.S., Wang Y.H. (2024). A multifunctional hydrogel fabricated by direct self-assembly of natural herbal small molecule mangiferin for treating diabetic wounds. ACS Appl. Mater. Interfaces.

[50] Zhang C.K., Zhou P.R., Li S.C., Zhang X.C., Xia Z.X., Rao Z.H., Ma X.M., Hu Y.J., Chen Y.C., Chen J.L. (2025). From hemostasis to angiogenesis: A self-healing hydrogel loaded with copper sulfide-based nanoenzyme for whole-process management of diabetic wounds. Biomater. Res..

[51] Chen Y., Xiang Y., Zhu T.H., Chen S.H., Du J., Luo J.J., Yan X.Y. (2022). A dZnONPs enhanced hybrid injectable photocrosslinked hydrogel for infected wounds treatment. Gels.

[52] Gao Q., Hu F.F., Chai Z.H., Zheng C.Y., Zhang W.H., Pu K., Yang Z.Y., Zhang Y.N., Ramrkrishna S., Wu X.L. (2025). Multifunctional hydrogel with mild photothermal properties enhances diabetic wound repair by targeting MRSA energy metabolism. J. Nanobiotechnol..

[53] Hu X.Q., Zhu J.Z., Hao Z.K., Tang L.T., Sun J., Sun W.R., Hu J.X., Wang P.Y., Basmadji N.P., Pedraz J.L. (2024). Renewable electroconductive hydrogels for accelerated diabetic wound healing and motion monitoring. Biomacromolecules.

[54] Zhang S.M., Ge G.R., Li W.H., Dong J.L., Hu X.L., Qin Y., Zhang P., Bai J.X., Zhang W.W., Su Z. (2025). Sr-MOF-based hydrogel promotes diabetic tissue regeneration through simultaneous antimicrobial and antiinflammatory properties. Mater. Today Bio.

[55] Wang H.B., Wang C.F., Wu S.L., Yan D.N., Huang C.H., Mao C.Y., Zheng Y.F., Liu H.P., Jin L.G., Zhu S.L. (2025). Accelerating interface NIR-induced charge transfer through Cu and black phosphorus modifying G-C_3_N_4_ for rapid healing of *Staphylococcus aureus* infected diabetic ulcer wounds. Small.

[56] Chen S.C., Qiu J.J., Chen S.H., Nie X.S., Zhao L.L., Wang F., Liu H.R., Liu X.Y. (2025). Zn ion-incorporated injected hydrogels with reactive oxygen species and glucose scavenging capacity for diabetic wound healing. Burn. Trauma.

[57] Yang X.L., Zhang Y., Huang C., Lu L., Chen J.Y., Weng Y.J. (2022). Biomimetic hydrogel scaffolds with copper peptide-functionalized RADA16 nanofiber improve wound healing in diabetes. Macromol. Biosci..

[58] Kim M.J., Ji H.B., Min C.H., Kim C.R., Han J.H., Kim S.N., Yoon S.B., Kwon E.J., Lee C.L., Choy Y.B. (2024). Fe-porphyrin cross-linked hydrogel for reactive oxygen species scavenging and oxygen generation in diabetic wounds. ACS Appl. Mater. Interfaces.

[59] Wang T.Y., Wen M.Y., Li N., Zhang L.B., Xue Y.M., Shang L. (2024). Marine-derived nanozyme-crosslinked self-adaptive hydrogels for programmed regulating the regeneration process. Adv. Funct. Mater..

[60] Geng X.R., Li W.J., Qu J.Q., Wang Q., Che T.J., Yan L.B., Cui H.L., Liu D.T., Qin S. (2025). Modified fish-skin-collagen-based hydrogels with antioxidant and antibacterial functions for diabetic wound healing. Adv. Healthc. Mater..

[61] Masood N., Ahmed R., Tariq M., Ahmed Z., Masoud M.S., Ali I., Asghar R., Andleeb A., Hasan A. (2019). Silver nanoparticle impregnated chitosan-PEG hydrogel enhances wound healing in diabetes induced rabbits. Int. J. Pharm..

[62] Ullah S., Hussain Z., Ullah I., Wang L., Mehmood S., Liu Y.S., Mansoorianfar M., Liu X.Z., Ma F.S., Pei R.J. (2023). Mussel bioinspired, silver-coated and insulin-loaded mesoporous polydopamine nanoparticles reinforced hyaluronate-based fibrous hydrogel for potential diabetic wound healing. Int. J. Biol. Macromol..

[63] Majie A., Saha R., Sarkar A., Bhowmik R., Karmakar S., Sharma V., Deokar K., Haque A.U., Tripathy S.S., Sarkar B. (2024). A novel chitosan-PEG hydrogel embedded with in situ silver nanoparticles of Clerodendrum glandulosum Lindl. extract: Evaluation of its in vivo diabetic wound healing properties using an image-guided machine learning model. Biomater. Sci..

[64] Kar A.K., Singh A., Singh D., Shraogi N., Verma R., Saji J., Jagdale P., Ghosh D., Patnaik S. (2022). Biopolymeric composite hydrogel loaded with silver NPs and epigallocatechin gallate (EGCG) effectively manages ROS for rapid wound healing in type II diabetic wounds. Int. J. Biol. Macromol..

[65] Qian Y.N., Zheng Y.J., Jin J., Wu X., Xu K.J., Dai M.L., Niu Q., Zheng H., He X.J., Shen J.L. (2022). Immunoregulation in diabetic wound repair with a photoenhanced glycyrrhizic acid hydrogel scaffold. Adv. Mater..

[66] Lv J., Liu D.Q., Ren Z.Y., Zhang Y., Wang B., Zhang X.H., Wang T.X., Guan Z.W., Yu K., Chu L.L. (2025). Double crosslinked hydrogels loaded with zinc-ellagic acid metal-organic frameworks combined with a mild heat stimulation promotes diabetic wound healing. Chem. Eng. J..

[67] Zhang J.F., Li J., Zhang Y., Zhao Y.S., Shen J., Du F.K., Chen Y., Li M.X., Wu X., Chen M.J. (2024). Bilayer hydrogel with a protective film and a regenerative hydrogel for effective diabetic wound treatment. Biomater. Sci..

[68] He J.H., Li Z.L., Wang J.X., Li T.Y., Chen J.Y., Duan X.L., Guo B.L. (2023). Photothermal antibacterial antioxidant conductive self-healing hydrogel with nitric oxide release accelerates diabetic wound healing. Compos. Part B-Eng..

[69] Wang X., Ma Z.H., He Y.J., Sun Y., Peng Q., Zhao M., Huang X.J., Lei L., Gu H., Gou K.J. (2025). Polydopamine nanoparticle-integrated smart bletilla striata polysaccharide hydrogel: Photothermal-triggered CO_2_ release for diabetic wound microenvironment modulation. Int. J. Nanomed..

[70] Wang P.H., Pu Y.J., Ren Y.H., Kong W.H., Xu L.L., Zhang W.J., Shi T.Q., Ma J.P., Li S., Tan X.Y. (2023). Enzyme-regulated NO programmed to release from hydrogel-forming microneedles with endogenous/photodynamic synergistic antibacterial for diabetic wound healing. Int. J. Biol. Macromol..

[71] Chen Q., Li S.Y., Zhao W.F., Zhao C.S. (2022). A rapid-triggered approach towards antibacterial hydrogel wound dressing with synergic photothermal and sterilization profiles. Biomater. Adv..

[72] Chu W.H., Wang P., Ma Z., Peng L., Guo C.Y., Fu Y.Q., Ding L.Z. (2023). Lupeol-loaded chitosan-Ag plus nanoparticle/sericin hydrogel accelerates wound healing and effectively inhibits bacterial infection. Int. J. Biol. Macromol..

[73] Glazar D., Stular D., Jerman I., Simoncic B., Tomsic B. (2025). Embedment of biosynthesised silver nanoparticles in polyNIPAAm/chitosan hydrogel for development of proactive smart textiles. Nanomaterials.

[74] Ren Y., Aierken A., Zhao L., Lin Z.S., Jiang J.J., Li B.L., Wang J.Y., Hua J.L., Tu Q. (2022). hUC-MSCs lyophilized powder loaded polysaccharide ulvan driven functional hydrogel for chronic diabetic wound healing. Carbohydr. Polym..

[75] Bei Z.W., Ye L., Tong Q., Ming Y., Yang T.Y., Zhu Y.Z., Zhang L.H., Li X.C., Deng H.Z., Liu J. (2024). Thermostimulated shrinking and adhesive hydrogel dressing for treating chronic diabetic wounds. Cell Rep. Phys. Sci..

[76] Su Q.Y., Tuo P., Li H.J., Mei H.Y., Yuan Y.H., Naeem A., Wang X.L. (2025). Phosphate-responsive nanocomposite hydrogel laden with umbilical cord blood exosomes and nanosilver accelerates diabetic and infectious wound healing. Arab. J. Chem..

[77] Kalirajan C., Palanisamy T. (2020). Bioengineered hybrid collagen scaffold tethered with silver-catechin nanocomposite modulates angiogenesis and TGF-*β* toward scarless healing in chronic deep second degree infected burns. Adv. Healthc. Mater..

[78] Deng M.Y., Zhang M., Huang R., Li H.Y., Lv W.X., Lin X.J., Huang R.Q., Wang Y. (2022). Diabetes immunity-modulated multifunctional hydrogel with cascade enzyme catalytic activity for bacterial wound treatment. Biomaterials.

[79] Yang J.R., Liu X.L., Tang Y., Bi X.Y., He H.Y., Sun X.H., Sui Q., Li D.L., Liu W.C., Liang D.D. (2024). Zeolitic imidazolate framework-8 nanocomposite hydrogels for diabetic wound healing. ACS Appl. Nano Mater..

[80] Liu C.F., Zeng H.J., Chen Z.Y., Ge Z.L., Wang B., Liu B., Fan Z.J. (2022). Sprayable methacrylic anhydride-modified gelatin hydrogel combined with bionic neutrophils nanoparticles for scar-free wound healing of diabetes mellitus. Int. J. Biol. Macromol..

[81] Wei W.L., Zheng J.Y., Zhong Z.W., Ding W., Huang Y.L., Liu Z.R., Chen X.Y., Yang W.Y., Shi S., Jin B. (2025). An antibacterial and anti-inflammatory hemostatic adhesive with dual Zn^2+^ and NO release for the neurovascular electrostimulation of infected diabetic wounds. Adv. Healthc. Mater..

[82] Chen T.L., Zhang X.L., Zhou S.Z., Fang D., Luo D.H., Ran Z.Y., Liu Y.C., Xu C., Cao J.H., Li X.D. (2025). A versatile and double cross-linked hydrogel with potent antibacterial and immunomodulatory Zn@Met nanocomplexes for enhanced diabetic-infected wound healing. Chem. Eng. J..

[83] Yang X., Duan X.M., Zhu L., Zhao X., Zhang S.R., Ni S., Wu S., Han Z.Q., Deng W.Q., Sun D. (2025). Carbon-dot-Zn^2+^ assembly hybrid multifunctional hydrogels for synergistically monitoring local glucose and repairing diabetic wound. ACS Appl. Mater. Interfaces.

[84] Wang X.X., Li H.S., Zhou M., Ling G.X., Zhang P. (2025). Smart bilayer hydrogels based on chitosan and its derivatives for wound repair and visual detection of blood glucose by triggering a cascade enzyme system. Int. J. Biol. Macromol..

[85] Guo L.Q., Lan J.X., Li J.H., Song Y.B., Wang X.L., Zhao Y.S., Yuan Y. (2024). A novel bola-molecular self-assembling hydrogel for enhancing diabetic wound healing. J. Colloid Interface Sci..

[86] Xiao J.S., Chen S.Y., Yi J., Zhang H.F., Ameer G.A. (2017). A cooperative copper metal-organic framework-hydrogel system improves wound healing in diabetes. Adv. Funct. Mater..

[87] Liu S., Yu J., Zhang Q.F., Lu H.T., Qiu X.P., Zhou D.F., Qi Y.X., Huang Y.B. (2020). Dual cross-linked HHA hydrogel supplies and regulates MΦ2 for synergistic improvement of immunocompromise and impaired angiogenesis to enhance diabetic chronic wound healing. Biomacromolecules.

[88] Li N., Ma Q., Xu L.Y., Wang Y., Zhang L., Jiang Y.G., Liu H.Z. (2024). Polydopamine reduced graphene oxide/chitosan-based hydrogel for the therapy of diabetic wound. Mater. Today Commun..

[89] Jiang F., Su Y., Zhao T.E., Ren R.Y., Chi Z., Liu C.G. (2024). An injectable hydrogel of enteromorpha polysaccharide/gelatin-derivatives inspired by siderophores and biocatalysis for addressing all phases of chronic diabetic wound healing. Chem. Eng. J..

[90] Han N., Xu Z.Y., Cui C.Y., Li Y., Zhang D.F., Xiao M., Fan C.C., Wu T.L., Yang J.H., Liu W.G. (2020). A Fe^3+^-crosslinked pyrogallol-tethered gelatin adhesive hydrogel with antibacterial activity for wound healing. Biomater. Sci..

[91] Lin X.J., Zhang M., Lv W.X., Li J., Huang R., Wang Y. (2024). Engineering carbon nanotube-based photoactive COF to synergistically arm a multifunctional antibacterial hydrogel. Adv. Funct. Mater..

[92] Wang R.Y., Qi Y.C., Liu W.C., Cheng Z.Q., Li W., Zhu H.Y., Zhao Y., Han J.H. (2025). Multifunctional gastrodia elata polysaccharide-based triple-network hydrogel promotes *Staphylococcus aureus* infected diabetes wound. Carbohydr. Polym..

[93] Xu Z., Liu Y.J., Ma R., Chen J., Qiu J.M., Du S., Li C.C., Wu Z.H., Yang X.F., Chen Z.B. (2022). Thermosensitive hydrogel incorporating prussian blue nanoparticles promotes diabetic wound healing *via* ROS scavenging and mitochondrial function restoration. ACS Appl. Mater. Interfaces.

[94] Yu T.T., Zhang J.M., Lai J.W., Deng M.J., Zhou Z.Y., Xia Z.B., Zhong C.Y., Feng X.Y., Hu Y.M., Guo X.R. (2025). Prussian blue nanohybrid hydrogel combined with specific far-infrared based on graphene devices for promoting diabetic wound healing. Mater. Des..

[95] Dong H., Li J., Huang X.Y., Liu H.T., Gui R. (2023). Platelet-membrane camouflaged cerium nanoparticle-embedded gelatin methacryloyl hydrogel for accelerated diabetic wound healing. Int. J. Biol. Macromol..

[96] Zhao S.H., Ling J.H., Wang N., Ouyang X.K. (2024). Cerium dioxide nanozyme doped hybrid hydrogel with antioxidant and antibacterial abilities for promoting diabetic wound healing. Chem. Eng. J..

[97] Augustine R., Zahid A.A., Hasan A., Dalvi Y.B., Jacob J. (2021). Cerium oxide nanoparticle-loaded gelatin methacryloyl hydrogel wound-healing patch with free radical scavenging activity. ACS Biomater. Sci. Eng..

[98] Ullah A., Al Mamun A., Zaidi M.B., Roome T., Hasan A. (2023). A calcium peroxide incorporated oxygen releasing chitosan-PVA patch for diabetic wound healing. Biomed. Pharmacother..

[99] Hu M.R., Li Z.Y., Liu Y., Feng Y.Q., Wang Z.Y., Huang R.F., Li L., Huang X.P., Shao Q., Lin W.Q. (2024). Multifunctional hydrogel of recombinant humanized collagen loaded with MSCs and MnO_2_ accelerates chronic diabetic wound healing. ACS Biomater. Sci. Eng..

[100] Jiang N., Liu X.W., Sui B.Y., Wang J.L., Liu X., Zhang Z. (2024). Using hybrid MnO_2_-Au nanoflowers to accelerate ROS scavenging and wound healing in diabetes. Pharmaceutics.

[101] Chen L.P., Wang X.Y., Ren M.J., Wang Y., Zhao J.M., Qiang T.T., Dong L.Y., Wang X.H. (2024). Promoting the healing of infected diabetic wound by nanozyme-containing hydrogel with anti-bacterial inflammation suppressing, ROS-scavenging and oxygen-generating properties. J. Biomed. Mater. Res. Part B.

[102] Li Q.L., Dong M.D., Han Q.Q., Zhang Y.J., Yang D.Z., Wei D.Q., Yang Y.L. (2024). Enhancing diabetic wound healing with a pH-responsive nanozyme hydrogel featuring multi-enzyme-like activities and oxygen self-supply. J. Control. Release.

[103] Su J.W., Dong X.Z., Xu C., Wang Z., Liu C.J., Yang H.J., Zhang D., Yu A.X. (2025). ROS-responsive hydrogel enables drug/ion/gas co-delivery for improving survival of multi-territory perforator flap in diabetes. Adv. Funct. Mater..

[104] Li Y.H., Zhang Q., Wang Q.B., Wang J.P., Zhang X.M., Yin C.X. (2025). Nanoscale and hollow inorganic sulfide@porous organic network doped intelligent hydrogels for accelerating diabetes wound therapeutics. Sci. China Chem..

[105] Sun Y., Zhu Y.N., Si J.H., Zhang R.K., Ji Y.L., Fan J.J., Dong Y.Z. (2025). Glucose-activated nanozyme hydrogels for microenvironment modulation *via* cascade reaction in diabetic wound. Chin. Chem. Lett..

[106] Wang Z., Ou X.L., Guan L., Li X.C., Liu A.A., Li L., Zvyagin A., Qu W.R., Yang B., Lin Q. (2023). Pomegranate-inspired multifunctional nanocomposite wound dressing for intelligent self-monitoring and promoting diabetic wound healing. Biosens. Bioelectron..

[107] Pu Y.J., Wang P.H., Yang R., Tan X.Y., Shi T.Q., Ma J.P., Xue W.L., Chi B. (2022). Bio-fabricated nanocomposite hydrogel with ROS scavenging and local oxygenation accelerates diabetic wound healing. J. Mater. Chem. B.

[108] Hu X.L., He J., Qiao L., Wang C., Wang Y., Yu R.X., Xu W., Wang F., Yang S.H., Zhang X.C. (2024). Multifunctional dual network hydrogel loaded with novel tea polyphenol magnesium nanoparticles accelerates wound repair of MRSA infected diabetes. Adv. Funct. Mater..

[109] Wang Z.G., Li W., Fan Y.Z., Xiao C.R., Shi Z.F., Chang Y.B., Liang G.Y., Liu C.L., Zhu Z.R., Yu P. (2024). Localized surface plasmon resonance-enhanced photocatalytic antibacterial of in situ sprayed 0D/2D heterojunction composite hydrogel for treating diabetic wound. Adv. Healthc. Mater..

[110] Zhang K.Y., Zhu J.J., Sun W.C., Zhang Y., Li W.J., Wang Y., Zhou C.Y., He Y.N., Qin J.L. (2025). Antibacterial betaine modified chitosan-based hydrogel with angiogenic property for photothermal enhanced diabetic wound repairing. Carbohydr. Polym..

[111] Ordeghan A.N., Khayatan D., Saki M.R., Alam M., Abbasi K., Shirvani H., Yazdanian M., Soufdoost R.S., Raad H.T., Karami A. (2022). The wound healing effect of nanoclay, collagen, and tadalafil in diabetic rats: An in vivo study. Adv. Mater. Sci. Eng..

[112] Luo Q., Yan C.Y., Ma R.T., Dai L., Shu W.J., Asghar A., Jia Z.G., Zhu X.L., Yu S. (2025). E-PL/MnO_2_ nanozymes/gellan gum/hyaluronic acid-based multifunctional hydrogel to promote diabetic wound healing. Int. J. Biol. Macromol..

[113] Yao H.L., You X.Y., Wu S.Q., Wang Y.H., Hu D., Ma Y.S., Luo J., Qiu J., Zhou L.H. (2025). Remolding waste liquid from the zeolite synthesis process into wrinkled dressings for diabetic wound therapeutics with immunomodulation. Energy Environ. Mater..

[114] Zhang M., Fu X., Wang Y., Qi C., Zhao Z., Huang B., Zhou M., Lin Y.F. (2025). A sprayable nucleic acid hydrogel for diabetic wound healing *via* immunomodulation and angiogenesis. Small.

[115] Zhao D., Tang X.Y., Chen X., Ma H.S., Zhong H.Y., Wu H., Yang L., Tang J., Sun Q., Gao S.J.Y. (2025). Thermoelectric bionic skin promotes diabetic wound healing by restoring bioelectric field microenvironment. Adv. Funct. Mater..

[116] Zakrzeska A., Krasinska N., Kitlas P. (2024). Effects of *Phallus impudicus* extract for accelerating hard-healing wounds in diabetes-induced rats. Acta Pol. Pharm..

[117] Hu B., Gao M.Z., Boakye-Yiadom K.O., Ho W., Yu W., Xu X.Y., Zhang X.Q. (2021). An intrinsically bioactive hydrogel with on-demand drug release behaviors for diabetic wound healing. Bioact. Mater..

[118] Zhou B., Zhang C., Dai S., Zhao J., Li H.Y., Peng Y.B., Chu Y.F., Chen Z., Qin H.T., Zeng H. (2025). Injectable cinnamaldehyde-loaded ZIF-8/Gallic Acid-Grafted gelatin hydrogel for enhanced angiogenesis and skin regeneration in diabetic wound healing. Front. Bioeng. Biotech..

[119] Bai Q.Y., Wang Y.R., Duan L.Y., Xu X.M., Hu Y.S., Yang Y., Zhang L., Liu Z.P., Bao H.H., Liu T.L. (2021). Cu-Doped-ZnO nanocrystals induce hepatocyte autophagy by oxidative stress pathway. Nanomaterials.

